# The Role of Ethnic Origin on Psychosocial Health in Portugal: An Examination of Risk and Protective Factors

**DOI:** 10.3390/healthcare13233071

**Published:** 2025-11-26

**Authors:** Jóni Ledo, Madalena Cruz, Henrique Pereira, Iara do Nascimento Teixeira, Guilherme Welter Wendt, Felipe Alckmin-Carvalho, Catarina Oliveira

**Affiliations:** 1 Department of Psychology and Education, Faculty of Social and Human Sciences, University of Beira Interior, Campus IV, 6200-209 Covilhã, Portugal; joni.ledo@ubi.pt (J.L.); madalena.cruz@ubi.pt (M.C.); hpereira@ubi.pt (H.P.); felipe.carvalho@ubi.pt (F.A.-C.); 2 Center for Research in Sport, Health, and Human Development (CIDESD), 5001-801 Vila Real, Portugal; 3 Foundation for Science and Technology, 1249-074 Lisbon, Portugal; 4 Department of Psychology, University of Minho, 4710-057 Braga, Portugal; iara.teixeira@ubi.pt; 5 Department of Medical Sciences, Postgraduate Program in Applied Health Sciences, Western Paraná State University, Francisco Beltrão 85601, Brazil; 6 Vila Real District Center of EAPN—European Anti-Poverty Network/Portugal (EAPN Portugal), 5000-260 Vila Real, Portugal; vilareal@eapn.pt

**Keywords:** social discrimination, psychological distress, resilience, social support, ethnicity, health inequities

## Abstract

Background: Social inequalities and vulnerability associated with ethnic and social minority status are risk factors for health inequities. Objective: To assess associations between psychosocial health, social discrimination, perceived social support, and resilience among people living in Portugal based on ethnic origin. Method: This is an observational and cross-sectional study carried out with 756 individuals aged between 18 and 84 (Mean = 39.3; Standard deviation = 13.79). The sample was probabilistic, and participants were recruited through convenience sampling, on online platforms. Participants responded to the Brief Symptom Inventory, Everyday Discrimination Scale, Multidimensional Scale of Perceived Social Support, Connor–Davidson Resilience Scale, and a sociodemographic questionnaire. Participants were divided into two groups, the first consisting of white Portuguese of European origin (majority group, *n* = 609, 80.56%) and the second consisting of black individuals, Afro-descendants, Roma, and Portuguese-Roma (minority group, *n* = 147, 19.44%). Results: Compared to the majority group, the minority group experienced greater structural disadvantages, including lower educational attainment, higher unemployment, and lower income, as well as significantly higher scores for psychological distress, social discrimination, perceived social support, and resilience. Regression analyses revealed that belonging to an ethnic minority predicts psychological distress, even when controlling for other variables. Social discrimination was found to be an important risk factor for psychological distress, while perceived social support and resilience were found to be protective factors. Conclusions: Our results provide preliminary evidence for developing public social policies to care for ethnic minority groups living in Portugal. Further, findings highlight the high frequency of discrimination reported by this group and its associated mental health problems, which underscore the importance of investing in anti-discrimination campaigns, establishing formal and informal social support mechanisms, and developing social strategies to empower and increase the resilience of these minority groups in Portugal.

## 1. Introduction

A combination of individual, systemic, and structural factors contributes to the emergence and maintenance of inequalities associated with ethnic discrimination in healthcare [1,2,3,4,5,6,7,8,9,10,11,12,13,14,15]. The Social Determinants of Health (SDH) model states that health and well-being are the result of a broad set of social, economic, and environmental factors. According to the definition, SDH corresponds to the conditions in which an individual is born, grows, works, lives, and ages, and to the factors that influence all conditions of daily life, including the social and physical environment, health services, and structural and social factors [5].

Equally, mental health is a central public health issue, yet it is well known that social inequalities and social injustice associated with ethnic minority status continue to generate strong inequities at all levels, with impacts on overall health [16,17]. Indeed, belonging to an ethnic minority constitutes a risk factor that increases the likelihood of having an undiagnosed and untreated mental illness [18,19,20] and greater difficulty or impediment in accessing health services or therapeutic resources [3,5]. Further, risks linked to minority status lower the chances of even having a mental disorder recognized when compared to majority ethnic groups [21]. This inequality has significant costs to health and equity, both for the individual and for society [6,7,8].

In this study, we focus on the concept of “perceived discrimination,” which is defined as an individual’s perception of being treated unfairly based on their minority social status [22]. This concept encompasses not only the episode of discrimination itself, but also the interpretation that the individual exposed to discrimination attributes to violence, and the narrative developed from this perception [23]. Discrimination can be perceived as unidimensional or intersectional, meaning that a minority group may attribute discrimination to a single sociodemographic characteristic, such as ethnicity or gender, or, on the other hand, may recognize the overlap of several socially marginalized characteristics and their effects in terms of greater exposure to prejudice and stigma [24,25]. Researchers have studied the relationship between perceived discrimination and various risk factors, including economic factors such as unemployment and financial status [26,27] and social factors such as identity and social capital [24,28].

There is also abundant evidence linking perceived discrimination to health and well-being indicators [23,29]. For example, studies report correlations with various mental health problems, such as anxiety and depression [30,31,32], physical health, such as obesity and cardiovascular problems [23,24,25,26,27,28,29,30,31,32,33,34], oral health deficits [35,36,37], and the use of maladaptive coping strategies [38]. A systematic review with meta-analysis found that the perception of discrimination produces lasting and pervasive effects on both physical health (r = −0.15, 95% CI = −0.22, −0.07) and mental health (r = −0.20, 95% CI = −0.24, −0.16). In addition to producing chronic stress (r = 0.11, with a 95% CI from 0.18 to 0.05), it is also directly associated with reduced adherence to healthy behaviors and/or increased frequency of behaviors harmful to health (r = 0.18, with a 95% CI from 0.22 to 0.15), such as tobacco, alcohol, and other drug abuse [39].

Ethnic discrimination has been strongly associated with high levels of psychological distress [40], a concept that can be defined as a set of symptoms and difficulties such as anxiety, depression, social dysfunction, and difficulty managing daily activities [41,42]. It constitutes an important public health problem that affects quality of life [43,44]. Studies have identified some vulnerability factors that increase the likelihood of experiencing distress, such as being female, having low educational attainment, adverse economic situations, suffering from obesity and chronic diseases, and mental health problems [45,46]. On the other hand, looking at protective factors, resilience and social support seem to have a positive effect on mental health and well-being. Resilience was conceptualized as a process-oriented trait that arises from the complex interactions that people develop in the socioecological systems in which they are embedded, involving dimensions such as attachment to place, leadership, community cohesion, robust social networks, and adaptive learning [47]. Keeping in mind an intersectional definition of resilience allows us to analyze in greater depth the intersection of identities, such as ethnicity, gender, and socioeconomic conditions, that influence both the effectiveness and availability of community resources. This perspective offers a more comprehensive view of resilience, particularly relevant for research in underrepresented populations. For example, in the context of migration, resilience is presented as a fundamental quality in coping with and resolving challenges, being crucial for successful integration, and consequently promoting psychological and social well-being, as well as life satisfaction and higher levels of overall health [48,49,50,51,52,53,54,55,56,57,58].

A second mechanism that the literature has pointed out as protective of the health of ethnic minority groups is the perception of social support, a concept that can be characterized in five forms of support: emotional (care and empathy), instrumental (material support), informational (advice and guidance), companionship (social and leisure activities), and validation (feedback for self-assessment) [59]. As well as the different sources from which this support may originate, such as family, friends, and community [59]. Like resilience, it acts as a protective factor against psychological distress, influencing the individual’s assessment of stressors, increasing the perception of control, and promoting the use of adaptive coping strategies, as well as promoting well-being [60,61]. Several studies have analyzed the connection between feelings of belonging to a social group and mental health in individuals belonging to ethnic minorities, finding that greater identification with the ethnic group is associated with greater life satisfaction and lower levels of psychological distress [62,63]. National identification and a sense of religious belonging have also been linked to increased satisfaction and decreased psychological distress [64,65,66,67].

Studies conducted with refugee populations have found that support from family, friends, groups, and the community in general, as well as a sense of belonging, are associated with higher levels of life satisfaction and psychological flourishing [57,58]. A study conducted with Brazilian and Cape Verdean immigrants in Portugal identified the perception of discrimination as a risk factor for psychological distress. On the other hand, the perception of social support and resilience were identified as protective factors [9]. Another study showed that experiences of discrimination in Roma and non-Roma communities living in socioeconomic vulnerability in Portugal are a significant challenge, with the Roma community reporting a significantly higher perception of discrimination compared to the non-Roma community. Despite this, no significant differences were found in terms of positive affect between the groups [10].

This study is relevant because it provides information that helps clarify how ethnic origin is associated with psychological distress through co-occurring psychosocial mechanisms that are understudied in Portugal. Although there is evidence that discrimination leads to poorer mental health, Portuguese research rarely models risk and protective processes together or tests their simultaneous contributions. This study simultaneously assesses perceived discrimination, a risk factor for mental health problems, and two theoretically grounded protective factors: perceived social support, a stress-buffering resource, and resilience, the capacity for adaptive recovery.

This approach addresses an important gap in the literature by examining whether and how protective resources operate in individuals who report discrimination and whether minority status remains a unique predictor of distress once these processes are considered. This integrated approach advances knowledge in the field in two ways. First, it moves beyond bivariate associations to a multivariate explanation that reflects the complexity of real-world at-risk populations, such as ethnic minorities. This allows for comparability with international evidence while generating context-specific estimates. Second, by identifying the relative weight of discrimination in relation to modifiable protective factors, the study provides actionable targets for prevention and care, informing mental health services and public policies aimed at mitigating inequalities among ethnic minorities living in Portugal.

Therefore, the objectives of the present study are: (1) To analyze possible differences in sociodemographic variables (income, education, and unemployment rate) and psychosocial variables (social discrimination, psychological distress, social support, and resilience) between groups of adult individuals belonging to ethnic majorities and minorities; (2) To assess possible correlations between social discrimination, psychological distress, perceived social support, and resilience among people living in Portugal based on ethnic origin; and (3) To assess the predictive effect of sociodemographic and psychosocial variables on the psychological distress of participants belonging to ethnic majorities and minorities.

The hypotheses of this study are: (H1) Participants from the ethnic minority group will have greater structural disadvantages and mental health indicators compared to the ethnic majority group; (H2) Positive and significant correlation between social discrimination and psychological distress, and significant and negative correlation between psychological distress and social support and resilience. We believe that these correlations will be found in both ethnic groups, but will be more pronounced in the minority ethnic group; and (H3) Socioeconomic vulnerability, social discrimination, and reduced scores for social support and resilience will be predictors of poorer mental health in the minority group.

## 2. Materials and Methods

This study has an observational and cross-sectional design and is quantitative in nature. We evaluated a sample of adults from different ethnic backgrounds living in Portugal. This study was funded by the Foundation for Science and Technology as part of the award of doctoral research grants in a non-academic environment to the first author (2023.01027.BDANA).

### 2.1. Participants and Procedures

This study involved participants aged 18 or over, residing in Portugal (*n* = 756). The sample was probabilistic and selected according to convenience criteria. The inclusion criteria for participants were age (≥18), ability to read and write, having access to the internet, tech-literacy skills and residential status in Portugal (least 6 months). The exclusion criteria included the fact that, even if residing in Portugal, their permanent residence was reported to be in another country. Data collection took place between February and May 2025.

The research protocol was developed by the first author and disseminated by the other authors, both personally and institutionally. Data collection was carried out online during the first half of 2025 through the dissemination of a link throughout the country. The dissemination was supported by the University of Beira Interior and the Vila Real district branch of the EAPN (European Anti-Poverty Network), a partner in this study. Participants were recruited via contacts with associations, universities, and Private Institutions of Social Solidarity with participation open to anyone willing to complete the questionnaire. As a result, it was not possible to calculate a response rate, which should be considered when interpreting the results. Completing the protocol took approximately 20 min. Participants did not receive any form of financial compensation.

### 2.2. Instruments

Sociodemographic questionnaire. Questionnaire developed for this research to characterize participants in terms of their age, gender, sexual orientation, marital status, education, whether participants have children, number of children, household, professional situation, and income.

Brief Symptom Inventory (BSI-18). This instrument assesses psychological symptoms in adults and contains three subscales: somatization, depression, and anxiety [68]. It consists of 18 items (e.g., Item: “Pains in heart or chest”), rated on a 5-point Likert scale from 0 (“Not at all”) to 4 (“Extremely”). It is a self-report questionnaire designed for participants to indicate the intensity with which they felt each symptom in the previous two weeks. The original scale has good internal consistency (α = 0.89), as does the version adapted and validated for the Portuguese population [69] with a Cronbach’s alpha of 0.80. In the present study, Cronbach’s alpha was 0.95.

Multidimensional Scale of Perceived Social Support (MSPSS). This scale assesses perceived social support [70]. The scale consists of 12 items (e.g., Item: “My family is willing to help me make decisions”), divided into 3 subscales and rated on a 7-point Likert scale, from 1 (completely disagree) to 7 (completely agree). The Portuguese version of the scale, translated and validated [71], retains the 12 items and the same scales. Both have good psychometric properties, in the original version (α = 0.88) as in the Portuguese version, ranging from 0.87 to 0.95. In this study, Cronbach’s alpha was 0.95.

Everyday Discrimination Scale—Portuguese Version (EDS-PT). The EDS is a scale that assesses experiences of everyday discrimination [72]. The scale consists of 8 items and is divided into two subscales: unfair treatment, and personal rejection (e.g., Item: “People act as if they think you are not smart”). Scoring is done on a Likert scale from 0 (never) to 5 (almost always, almost every day). At the end of the 8 items, respondents who answer that these experiences happen at least “sometimes” should mention the type of discrimination they have suffered, based on a list of reasons. The Portuguese version of the scale was translated and validated [73], maintaining the number of items and subscales of the original scale. The original version has very good psychometric properties (α = 0.88), as does the Portuguese version, with a Cronbach’s alpha ranging from 0.83 to 0.95. In this study, Cronbach’s alpha was 0.95.

Connor–Davidson Resilience Scale-10 (CD-RISC-10). This scale is a self-assessment questionnaire for evaluating resilience in individuals, regardless of whether or not they have a disorder. The scale has 10 items (e.g., Item: “I am able to adapt when changes occur”), divided into five factors: Flexibility (items 1 and 5), sense of self-efficacy (items 2, 4, and 9), regulating emotions (item 10), optimism (items 3, 6, and 8), and maintaining attention under stress (item 7). The rating is made on a 5-point Likert scale ranging from “not true” (0) to “true almost all the time” (4). The original scale has very good internal consistency (α = 0.89) and the version adapted for the Portuguese population has a Cronbach’s alpha of 0.87 [74]. In the present study, Cronbach’s alpha was 0.91.

### 2.3. Data Analysis

For data analysis, we used the IBM SPSS statistical program, version 29, setting the significance at 5% (*p* < 0.05). The Shapiro–Wilk test was performed to assess the normality of the distribution of the variables analyzed. Since the distribution met the normality criterion, we used parametric tests. Based on self-reported ethnic origin, two distinct groups were defined: Ethnic Majority (*n* = 609, 80.56%), which covers the majority of the Portuguese population (White/Portuguese white/Of European origin) and ethnic minorities (*n* = 147, 19.44%), which includes other communities residing in Portugal (Black/Black, Portuguese/Afro-descendant/African origin, Roma/Portuguese Roma and other). Although Roma are anthropologically considered Caucasian due to their origins and migration from northern India to Europe between the 11th and 15th centuries, they constitute a distinct minority in Portugal. Historically, Roma have faced severe social exclusion and discrimination, ranking among the most discriminated-against groups in Europe, with 96% living in situations of poverty risk and 51.3% of Roma in Portugal have experienced discrimination at some point in their lives [44]. The number of participants per ethnic group is quite uneven, which roughly reflects the distribution of the population in Portugal. According to the National Statistics Institute, 84.1% (majority) of people identify as White Portuguese and 15.9% as Non-white (minority) [75]. To outline the sociodemographic profile of the participants and the scores of the psychosocial variables investigated, we performed statistics such as simple frequency, percentage, mean, standard deviation, theoretical median, and minimum and maximum scores. To assess sociodemographic and psychosocial differences between majority and minority ethnic groups, we used Student’s *t*-test for independent samples. The effect size for group differences was calculated using Cohen’s d, interpreted as follows: values below 0.20 indicate a negligible effect; values between 0.20 and 0.49 represent a small effect; 0.50 to 0.79 indicate a medium effect; and values of 0.80 or above reflect a large effect [76]. To assess correlations between psychological distress, social discrimination, perceived social support, and resilience, we used Pearson’s correlation test. To analyze the predictive effect of independent variables on psychological distress, we performed linear regression with three models. We used G*Power, version 3.0, software to calculate the statistical power of the study. Cohen’s d values ranged from 0.447 to 1.798 in the total sample of 756. In this sense, the power achieved was approximately 99.9%.

### 2.4. Ethical Considerations

This research was approved by the Ethics Committee of the University of Beira Interior (Ref. no. CE-UBI-Pj-2023-051-ID1915). All authors of the scales used in this research were contacted and gave permission for their use. All participants were duly informed about the objectives of the study and signed the informed consent form. Anonymity and confidentiality were ensured, in accordance with the ethical principles established in the Declaration of Helsinki [77].

## 3. Results

This study had a sample of 756 participants (M = 39.3; SD = 13.79) residing in Portugal, of whom 276 (36.5%) identified as men, 476 (63%) as women, and 4 (0.5%) as another gender. The mean age of the majority ethnic group was 40.4 years (SD = 14.07) and that of the minority ethnic group was 34.5 years (SD = 11.42). Table 1 shows the general sociodemographic characteristics of the sample, separated by ethnic group (majority and minority).

As shown in Table 1, the groups are quite distinct in several sociodemographic characteristics, with participants belonging to the ethnic minority group having lower educational levels, a greater number of people living in the same household, a greater number of children, a higher percentage of unemployed people, and lower income, all of which differences are statistically significant with effect sizes (Cramer’s V) ranging from 0.12 to 0.52. In this case, for the variables gender and marital status, the effect is small; for the variables number of children, professional situation, and income, the effect is medium; and for the variables education and household, the effect is large. In an additional analysis using Student’s *t*-test, it was found that per capita income was significantly higher in the majority ethnic group (M = 1.70, SD = 0.46) compared to the minority group (M = 1.14, SD = 0.35), *p* < 0.001, d = 1.35.

### 3.1. Comparison Between Majority and Minority Ethnic Groups

Table 2 shows the results of Student’s *t*-test for comparing independent samples between the majority and minority communities. The results indicate that the minority community has significantly higher levels (*p* < 0.001) of psychological distress, greater social discrimination, perceived social support, and resilience.

### 3.2. Correlations Between Psychological Distress, Social Discrimination, Perceived Social Support, and Resilience

**Figure 1:**
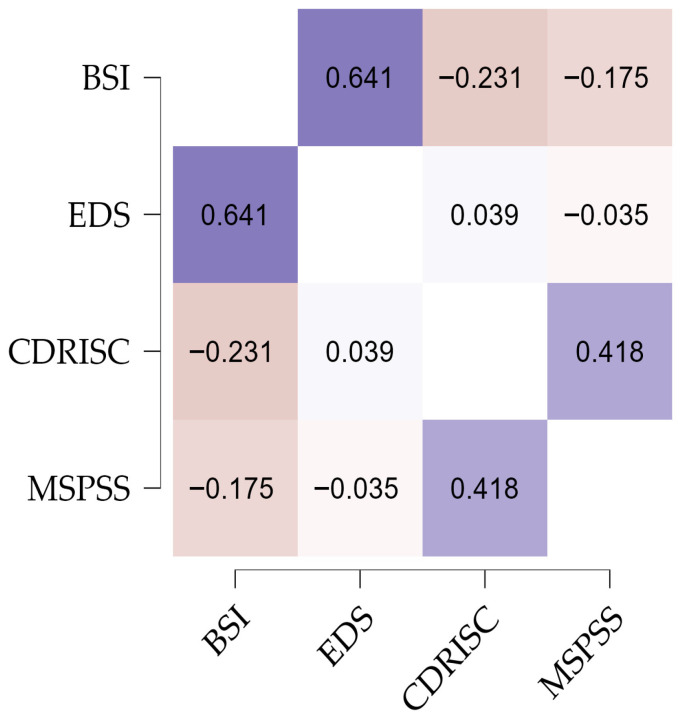
Correlations between the study variables. Notes. BSI: Brief Symptom Inventory; CD-RISC-10: Connor–Davidson Resilience Scale-10; EDS: Everyday Discrimination Scale—Portuguese Version; MSPSS: Multidimensional Scale of Perceived Social Support.

As shown in Figure 1, psychological distress showed a significant, moderate, positive correlation with social discrimination (0.641) and a significant, weak, negative correlation with perceived social support (−0.175) and resilience (−0.231). In the case of social discrimination, as it increases, psychological distress also increases. With regard to perceived social support and resilience, the correlation with psychological distress was significant, weak and negative, which means that as perceived social support and resilience increase, psychological distress decreases. The results also show a significant, moderate, and positive correlation between resilience and perceived social support (0.418).

### 3.3. Predictive Effect of Independent Variables on Psychological Distress

The results of the linear regression with the effect of sociodemographic variables and social discrimination, perceived social support, and resilience on the dependent variable psychological distress are depicted in Table 3. We created three predictive models: Model 1, with sociodemographic variables, Model 2, with the inclusion of the independent variables studied (i.e., social discrimination, perceived social support, and resilience), and Model 3, with the inclusion of the ethnicity variable. Model 1 explains 21.8% of the variance and shows that the higher the age, education, and income, the lower the psychological distress. It also shows that belonging to a sexual minority is a predictor of greater psychological distress. In Model 2, which explains 49.7% of the variance, with the inclusion of psychological variables and ethnicity, the total variance explained increases gradually, with significant results in all variables. In Model 2, only age and sexual orientation remain significant in the same sense, and the gender variable becomes significant, demonstrating that being male is a predictor of lower psychological distress. Social discrimination presents significant and negative results, that is, the higher the social discrimination, the higher the levels of psychological distress, with social discrimination acting here as a risk factor for mental health. In the case of perceived social support and resilience, the higher their values, the lower the psychological distress, indicating that these variables act as protective factors for psychosocial health. In Model 3, which explains 50.2% of the variance, we found that the significant variables remain unchanged and with the same sign, but the ethnicity variable is significant, showing that belonging to an ethnic minority is a predictor of greater psychological distress.

## 4. Discussion

The present study aimed to aid in improving care for at-risk populations. Particularly by exploring the correlates of social discrimination, perceived social support, and resilience among people living in Portugal based on majority or minority ethnic origin. We also sought to understand how sociodemographic and psychosocial variables predict psychological distress—a hallmark of vulnerability for overall incapacity and illness propension—while also comparing potential differences between those not at-risk (i.e., majority) and at-risk (i.e., minority ethnic).

The results confirm the proposed hypotheses, revealing relevant sociodemographic disparities between ethnic groups, with the at-risk, minority group displaying well-known signs of socioeconomic vulnerability. Hence, significant associations were found between psychosocial variables, highlighting the role of social discrimination as an important risk factor for psychological distress, while perceived social support and resilience emerge as protective factors. In addition, belonging to an ethnic minority was found to be a significant predictor of greater psychological distress, even after controlling other variables. These findings reinforce the importance of considering ethnicity as a central dimension in the analysis of psychosocial health in contexts of risk factors and impediments for healthcare.

The Social Determinants of Health (SDH) framework helps explain these findings by showing how health outcomes are shaped by the conditions people face throughout their lives. In our study, participants from ethnic minority backgrounds experienced greater structural disadvantages, including lower educational attainment, higher unemployment, and lower income, which are key elements of the SDH model [5]. These social determinants appear to have a direct contribution to this group’s heightened psychological distress. Evidence in other countries also points in the same direction. For example, a research study in the US found that unemployment, food insecurity, and housing instability are strongly interlinked with psychological distress among ethnic minorities [11].

Another review of ethnic minority adolescents reported that factors like acculturative stress and lower parental education raise mental health risks in these groups [12]. Together, these findings support our results and highlight that social discrimination, which is central to the SDH framework, is a strong predictor of psychological distress even after accounting for sociodemographic differences. To reduce ethnic disparities in mental health, we need to address the structural inequalities that influence risk and access to support, rather than focusing only on individual solutions.

Results are consistent with the growing body of evidence pointing to ethnic discrimination as a critical social determinant of psychological distress in minority populations. For example, a study of Sami and Kven communities in Norway found that ethnic discrimination was strongly associated with high levels of psychological distress, even when sociodemographic variables were controlled for [40]. Similarly, among Palestinian adolescents living in Israel, exposure to racial microaggressions in low-ethnic-density environments was found to be directly related to increased psychological distress [78]. Our study confirms this trend in the Portuguese context, identifying social discrimination as the strongest predictor and the major risk factors underpinning psychological distress, even after controlling for age, gender, sexual orientation, income, and education. Furthermore, belonging to an ethnic minority proved to be an independent predictor of psychological distress, a pattern also observed in Latin American populations in the United States, where the context of ethnic minority amplified the negative effects of discrimination and social exclusion on mental health [13]. An interesting finding in our study refers to the greater perception of social support and resilience among participants in the minority group, even in the face of greater psychosocial adversity. A similar pattern was observed in investigations conducted in the United States and China, countries where marginalized communities report more intense support in family and community networks as a strategy for coping with social exclusion [79,80]. However, even with these high protective resources, levels of psychological distress remain significantly higher in the minority group among our participants, suggesting that social support and resilience, while important, are not sufficient to counteract the cumulative impacts of the risks posit by racial and ethnic discrimination. Chronic exposure to discriminatory events can generate compounding effects of stress that exceeds an individual’s capacity for coping. Additionally, a study of British youth showed that personal and ethnic self-esteem acted as a partial mediator of the impact of discrimination on psychological distress, suggesting that strengthening collective identity may be an additional protective factor, although not fully compensatory [81].

In addition to psychosocial variables, the data from the present study might be important in implementing culturally sensitive healthcare practices by promoting social justice and awareness. The significant sociodemographic disparities between ethnic groups reveal power dynamics and help in explaining the higher levels of psychological distress among at-risk participants. Differences in education, employment, family composition, and income are significant: 44.9% of participants in the minority group were unemployed (vs. 12.2% in the majority group) and only 25.2% had a university education (vs. 71.8%).

These structural asymmetries are especially relevant, since poor socioeconomic conditions are widely recognized as central determinants of health. Low socioeconomic status is consistently associated with a higher occurrence and greater chronicity of mental health problems, as well as less seeking treatment and lower adherence to therapeutic regimens. The relationship between low socioeconomic status and poorer mental health indicators is mediated by chronic stressors, such as limited time, fewer leisure opportunities, greater exposure to violence, less access to health care, and experiences of stigma. Studies indicate that this association is likely bidirectional, in that poorer mental health indicators reduce the capacity to produce, generate income, and ascend socially, perpetuating inequalities. International evidence indicates that the protective effects of income and education are not distributed evenly across ethnic groups, especially in contexts marked by persistent structures of racial or ethnic discrimination [14,15].

For example, it has been demonstrated that, among Latino subgroups in the United States, psychological distress remained high even at reasonable levels of education or income, particularly among Puerto Ricans and Dominicans [82]. This pattern stems from a complex interaction between socioeconomic status, ethnicity, and experiences of discrimination. More broadly, social inequality does not operate in isolation but is intertwined with racial exclusion. Indeed, it has been observed that high-income black men in the United States had high levels of psychological distress, suggesting that structural racism is a risk factor regardless of socioeconomic status [83].

### 4.1. Limitations of the Study and Future Directions

We believe that the objectives set for this research were achieved, but it was not without limitations. The cross-sectional nature of the study does not allow us to infer causal relationships between the variables studied and their implications for psychosocial health of ethnic minorities. Therefore, longitudinal studies are needed to verify these causal relationships and see how the results vary over time. Furthermore, our sample was selected by convenience criteria and may not be representative of the ethnic minority population living in Portugal. Thus, these limitations suggest caution in generalizing our findings.

We did not control the participants’ sociodemographic variables, such as gender, age, sexual orientation, and family relationships that may impact psychosocial health. Moreover, as guided by the research goals, regression analyses sought to investigate factors independently linked with psychosocial distress. In this regard, it might be useful for future investigations to construct tentative hypotheses that direct analyses that focus on interactions between independent variables.

In addition, there are also relevant sociodemographic differences between groups in terms of education, with the majority community having higher levels of education. Education may impact on how they perceive the items and generate bias in the results. Online data collection is also a limitation, as older participants or those with low digital literacy are excluded. Furthermore, it is important to recognize the possible bias associated with the use of self-report instruments, which can influence the accuracy of responses, as well as the cultural specificity of the Portuguese context, in terms of social norms and values which limits the generalization of the results to other populations. The difference in the sample between the two groups, although representing the proportion of the Portuguese population, and grouping diverse minorities into one category are also a limitation. This approach obscures important heterogeneity across ethnic minority groups, such as differences in socioeconomic status, educational attainment, cultural experiences, and exposure to discrimination such as differences in socioeconomic status, educational attainment, cultural experiences, and exposure to discrimination. Consequently, this aggregation prevents detailed comparisons between the specific minority groups in the sample and their counterparts in national datasets, which disaggregate ethnicity into distinct categories. This study also fails to consider differences in health status or in access to quality of healthcare in general, factors that are important determinants of community well-being.

Future research should include them to better understand the complexity of health inequalities and examine these subgroups separately.

We also recommend broader and more representative sampling of ethnic minority communities across different regions of Portugal, including urban and rural municipalities. Probability-based designs with stratification and oversampling of Roma, Portuguese Roma, Afro-descendant and other under-represented groups, paired with post-stratification weights. This could provide valuable insights into the particularities of the relationship between discrimination and psychological distress within each ethnic minority group. Finally, we recommend conducting qualitative studies, based on in-depth clinical interviews, to understand the experiences of discrimination experienced by ethnic minorities, the respective psychosocial effects, and the narratives produced at the individual level about these experiences. We believe that an in-depth analysis of the perspective of individuals belonging to ethnic minorities can generate relevant clinical insights, especially for understanding the moderators of the relationship between discrimination and mental health problems, as well as insights relevant to public policies, which should be developed based on an assessment of the needs, facilitators, and barriers to the social integration of ethnic minorities in Portugal.

### 4.2. Practical Implications for Clinicians and Policymakers

Our results suggest that belonging to an ethnic minority, in combination with greater exposure to discrimination and socioeconomic disadvantage, is associated with greater psychological distress. The first implication is therefore systemic: public policies should reduce structural inequalities in areas such as professional and academic development opportunities, and access to decent work and housing. At the level of health services, the findings support the implementation of culturally sensitive adaptations to care. These include routine screening for experiences of discrimination and psychological distress in primary and mental healthcare, clear referral pathways, and the use of cultural and linguistic mediators, including community mediators from minority groups. Ongoing training of teams in cultural competence, implicit bias, person-centered communication, and social referrals to community resources that expand social support and access to educational, recreational, and cultural opportunities are also recommended.

At the community level, investment in community organizations to develop peer support programs, psychoeducational groups and mentoring networks that reinforce perceived social support is recommended. This is one of the protective factors identified in our study. Peer-led programs, e.g., health promoters, community agents, and Roma/Afro-descendant mediators, can provide guidance on navigating systems, improve health literacy, and refer individuals to social services. At the same time, communication campaigns co-created with communities can reduce stigma, increase knowledge of rights, and boost demand for services. At the clinical level, our findings suggest integrating resilience training and culturally adapted coping strategies into standard psychological interventions. This includes training in social skills such as emotional regulation and problem solving, as well as strengthening identity and belonging and taking a trauma- and stress-related discrimination-focused approach.

At the clinical level, our findings suggest that resilience training and culturally adapted coping strategies should be integrated into standard psychological interventions. This would involve training in social skills such as emotional regulation and problem solving, as well as strengthening identity and sense of belonging. It would also involve approaches focused on trauma and stress related to discrimination. Regulatory instruments, such as anti-discrimination protocols and safe reporting channels in schools, services and workplaces, should be accompanied by accountability mechanisms and compliance incentives, such as diversity targets and transparency reports. Crucially, administrative data and population surveys should collect, and report information disaggregated by ethnic origin to allow disparities and the impact of interventions to be assessed. Combined, these strategies will translate the findings of this study into concrete actions to mitigate risks, enhance protective factors and reduce inequalities among ethnic minorities in Portugal.

## 5. Conclusions

Belonging to an ethnic minority group was associated with social discrimination and psychological distress. In comparison with socially hegemonic group, we found that people from ethnic minorities have higher levels of psychological distress and social discrimination and higher levels of perceived social support and resilience. These results suggest that psychological distress and social discrimination may reflect key social determinants affecting the psychosocial health of these communities, and that perceived social support and resilience act as protective factors. Our results highlight that, although efforts are being made to combat discrimination and social inequalities, these phenomena are still very prevalent in Portugal and provide preliminary evidence for developing public social policies to care for ethnic minority groups living in this country. Additionally, our results underscore the importance of investing in anti-discrimination campaigns, establishing formal and informal social support mechanisms, and developing social strategies to empower and increase the resilience of these minority groups in Portugal.

## Figures and Tables

**Table 1:** Sociodemographic characteristics of participants.

		Ethnic Majority (*n* = 609, 80.56%)		Ethnic Minority (*n* = 147, 19.44%)		Total Sample (*n* = 756, 100%)				
		N	%	N	%	N	%	χ^2^	*p*	Cramer’s V
Gender	Men	183	30	93	63.3	276	36.5	56,690	<0.001	0.274
	Women	422	66.3	54	36.7	476	63			
Sexual orientation	Straight	540	88.8	127	86.4	667	88.2	0.590	0.442	0.028
	LGBTQIA+	69	11.3	20	13.6	89	11.8			
Marital Status	Single/Not dating	139	22.8	32	21.8	171	22.6	10.95	0.027	0.120
	Single/Dating	140	23	34	23.1	174	23			
	Married/Cohabitation	271	44.5	75	51	346	45.8			
	Divorced/Separated	51	8.4	2	1.4	53	7			
	Widow	8	1.3	4	2.7	12	1.6			
Children	No	312	51.2	68	46.3	380	50.3	1171	0.279	0.039
	Yes	297	48.8	79	53.7	376	49.7			
Number of children	1	125	41.8	5	6.4	130	34.6	78.64	0.001	0.459
	2	130	43.5	30	38.5	160	42.6			
	3	27	9	27	34.6	54	14.4			
	4	8	2.7	12	15.4	20	5.3			
	5 or more	8	2.7	4	5.1	12	3.2			
Education	Up to 4 years	3	0.5	8	5.4	11	1.5	187.66	0.001	0.498
	Up to 6 years	9	1.5	20	13.6	29	3.8			
	Up to 9 years	25	4.1	42	28.6	67	8.9			
	Up to 12 years	135	22.2	40	27.2	175	23.1			
	Degree	235	38.6	20	13.6	255	33.7			
	Master’s Degree	201	33	17	11.6	218	28.8			
	Other	1	0.2			1	0.1			
Household	1	84	13.8	9	6.1	93	12.3	203.34	0.001	0.519
	2	176	28.9	20	13.6	196	25.9			
	3	176	28.9	10	6.8	186	24.6			
	4	138	22.7	35	23.8	173	22.9			
	5	27	4.4	45	30.6	72	9.5			
	More than 5 people	8	1.3	28	19	36	4.8			
Professional situation	Unemployed	74	12.2	66	44.9	140	18.5	118.28	0.001	0.396
	Employed	330	54.2	25	17	355	47			
	Self-employed worker	48	7.9	3	2	51	6.7			
	Worker/student	28	4.6	11	7.5	39	5.2			
	Student	106	17.4	30	20.4	136	18			
	Retired	18	3	11	7.5	29	3.8			
	Other	5	0.8	1	0.7	6	0.8			
Income	Up to 522.50 euros	46	7.6	38	25.9	84	11.1	175.19	0.001	0.481
	From 522.50 to 1045 euros	74	12.2	66	44.9	140	18.5			
	From 1045 to 1567.5 euros	101	16.6	31	21.1	132	17.5			
	From 1567.5 to 2090 euros	115	18.9	3	2	118	15.6			
	From 2090 to 2612.5 euros	98	16.1	2	1.4	100	13.2			
	From 2612.5 to 3135 euros	61	10	4	2.7	65	8.6			
	From 3135 to 3657.5 euros	37	6.1	1	0.7	38	5			
	More than 3657.5 euros	77	12.6	2	1.4	79	10.4			

**Table 2:** Comparison Between majority and minority ethnic groups.

Variables	Ethnicity	Mean	SD	*p*	Cohen’s d
Psychological distress	Majority	0.82	0.69	<0.001	0.997
	Minority	1.57	0.80		
Social discrimination	Majority	0.81	0.81	<0.001	1.798
	Minority	2.62	1.17		
Perceived social support	Majority	5.58	1.17	<0.001	0.447
	Minority	6.09	1.09		
Resilience	Majority	2.64	0.73	<0.001	0.84
	Minority	3.17	0.52		

**Table 3:** Analysis of linear regression as predictors of psychological distress.

	Model 1			Model 2			Model 3		
Psychological Distress	B	SEB	β	B	SEB	β	B	SEB	β
Gender	0.041	0.935	0.001	−1.504	0.766	−0.054 *	−1698	0.767	−0.061 *
Sexual orientation	6178	1474	0.144 **	2628	1203	0.061 *	2651	1198	0.062 *
Age	−0.203	0.036	−0.202 **	−0.091	0.030	−0.090 **	−0.087	0.030	−0.086
Education	−2.205	0.472	−0.187 **	−0.342	0.398	−0.029	−0.257	0.398	−0.022
Household	0.672	0.377	0.065	−0.037	0.312	−0.004	−0.135	0.313	−0.013
Per capita income	−4906	1146	−0.175 **	−1010	0.946	−0.036	−0.605	0.955	−0.022
Social discrimination				7.253	0.397	0.601 **	6717	0.446	0.556 **
Perceived social support				−0.705	0.347	−0.060 **	−0.811	0.348	−0.069 *
Resilience				−3898	0.565	−0.204 **	−4295	0.583	−0.224 **
Ethnicity							3412	1312	0.098 **
R^2^	0.218			0.497			0.502		
Z	34.73			81.99			75.04		

B = unstandardized coefficient; SEB = standard error; β = standardized coefficient; R^2^ = coefficient of determination; Z = z-score; *p*-value = * <0.05; ** <0.01.

## Data Availability

Data can be obtained from the authors upon reasonable request.
